# Left bundle branch area pacing in patients with transthyretin cardiac amyloidosis: a case series

**DOI:** 10.1093/ehjcr/ytae677

**Published:** 2025-01-15

**Authors:** Keisuke Miyajima, Wakaba Kobayashi, Shogo Hakamata, Yasukazu Takazawa, Yoshitaka Kawaguchi, Yasushi Wakabayashi, Yuichiro Maekawa

**Affiliations:** Department of Cardiology, Seirei Mikatahara General Hospital, 3453 Mikatahara-cho, Kita-ward, Hamamatsu, Shizuoka 433-8558, Japan; Department of Cardiology, Seirei Mikatahara General Hospital, 3453 Mikatahara-cho, Kita-ward, Hamamatsu, Shizuoka 433-8558, Japan; Department of Cardiology, Seirei Mikatahara General Hospital, 3453 Mikatahara-cho, Kita-ward, Hamamatsu, Shizuoka 433-8558, Japan; Department of Cardiology, Seirei Mikatahara General Hospital, 3453 Mikatahara-cho, Kita-ward, Hamamatsu, Shizuoka 433-8558, Japan; Department of Cardiology, Seirei Mikatahara General Hospital, 3453 Mikatahara-cho, Kita-ward, Hamamatsu, Shizuoka 433-8558, Japan; Department of Cardiology, Seirei Mikatahara General Hospital, 3453 Mikatahara-cho, Kita-ward, Hamamatsu, Shizuoka 433-8558, Japan; Division of Cardiology, Internal Medicine III, Hamamatsu University School of Medicine, Hamamatsu, Shizuoka 431-3192, Japan

**Keywords:** Transthyretin cardiac amyloidosis, Atrioventricular block, Left bundle branch area pacing, Ventricular septal hypertrophy, Physiological pacing, Heart failure, Case series

## Abstract

**Background:**

Transthyretin cardiac amyloidosis is associated with various arrhythmias, including atrioventricular block. Despite this correlation, established treatments for transthyretin cardiac amyloidosis-associated arrhythmias are lacking. Left bundle branch area pacing is a promising physiological pacing technique.

**Case summary:**

This case series describes three instances of successful left bundle branch area pacing in patients with transthyretin cardiac amyloidosis presenting with atrioventricular block. Despite significant ventricular septal hypertrophy across all cases, left bundle branch area pacing was implemented effectively without complications.

**Discussion:**

Traditional pacing strategies in transthyretin cardiac amyloidosis, such as right ventricular pacing, have been associated with a reduced left ventricular ejection fraction and worsening heart failure. Although biventricular pacing has been explored, the supporting evidence remains limited and inconclusive. Recent studies have suggested that left bundle branch area pacing poses a lower risk of inducing heart failure than biventricular pacing. Our findings support the safety and efficacy of the left bundle branch area pacing in patients with transthyretin cardiac amyloidosis-related atrioventricular blocks and underscore its viability as a pacing strategy.

**Learning points**

- Left bundle branch area pacing (LBBAP) can be effectively utilized in patients with transthyretin cardiac amyloidosis (ATTR-CA) presenting with atrioventricular block, demonstrating improved electrical and mechanical synchrony without significant complications.
- Familiarity with electrophysiological insights and the use of various delivery catheters are essential in LBBAP for ATTR-CA, enhancing the success and safety of the procedure.

## Introduction

Transthyretin cardiac amyloidosis (ATTR-CA) is associated with various arrhythmias, including atrioventricular (AV) block.^1^ However, an optimal pacing method for the AV blocks related to ATTR-CA has not yet been established. Right ventricular (RV) pacing (RVP) can induce left ventricular (LV) dyssynchrony and reduce cardiac output due to asynchronous ventricular activation.^2,3^ While biventricular pacing (BiVP) is commonly used to preserve LV synchrony in patients with reduced LV ejection fraction (LVEF), many patients with ATTR-CA have preserved LVEF and do not meet standard criteria for cardiac resynchronization therapy with BiVP. Consequently, BiVP is often not indicated despite significant electrical conduction delays seen in ATTR-CA patients.

Left bundle branch area pacing (LBBAP) has recently been established as a useful physiological alternative to RVP, showing promise in patients with cardiac conditions such as ATTR-CA.^4^ Some studies have reported that LBBAP reduces electrical and mechanical dyssynchrony compared with RVP.^5^ Moreover, some reports suggest the superiority of LBBAP over BiVP in terms of QRS narrowing, improvement in LVEF, and reduction in heart failure rates.^6,7^

## Summary figure

ATTR-CA, transthyretin cardiac amyloidosis; AV, atrioventricular; BNP, B-type natriuretic peptide; CLBBB, complete left bundle branch block; CRBBB, complete right bundle branch block; ECG, electrocardiography; LBBAP, left bundle branch area pacing; LVEF, left ventricular ejection fraction; V6RWPT, V6 R-wave peak time.

**Figure:**
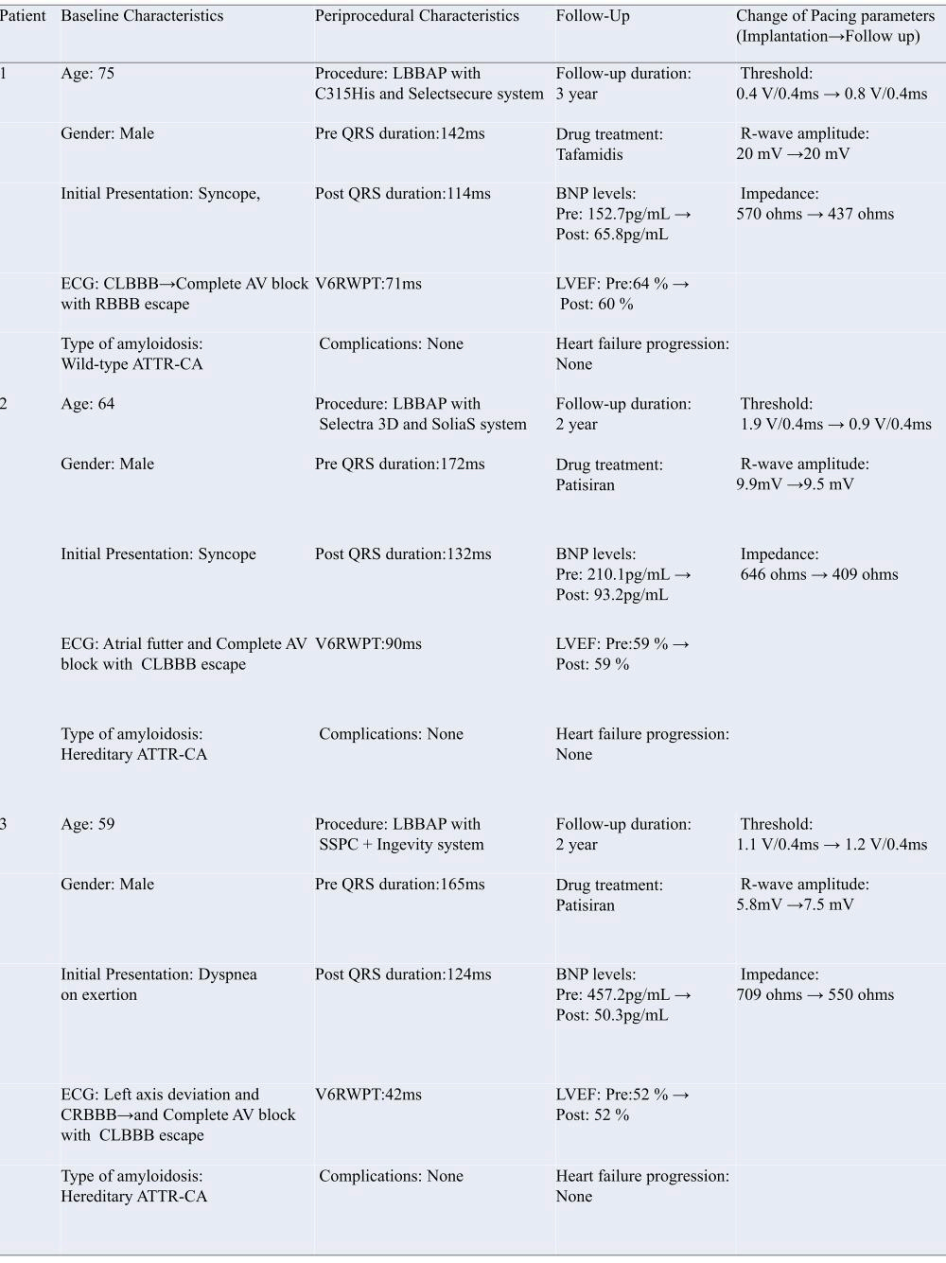


## Patient 1

**Figure 1:**
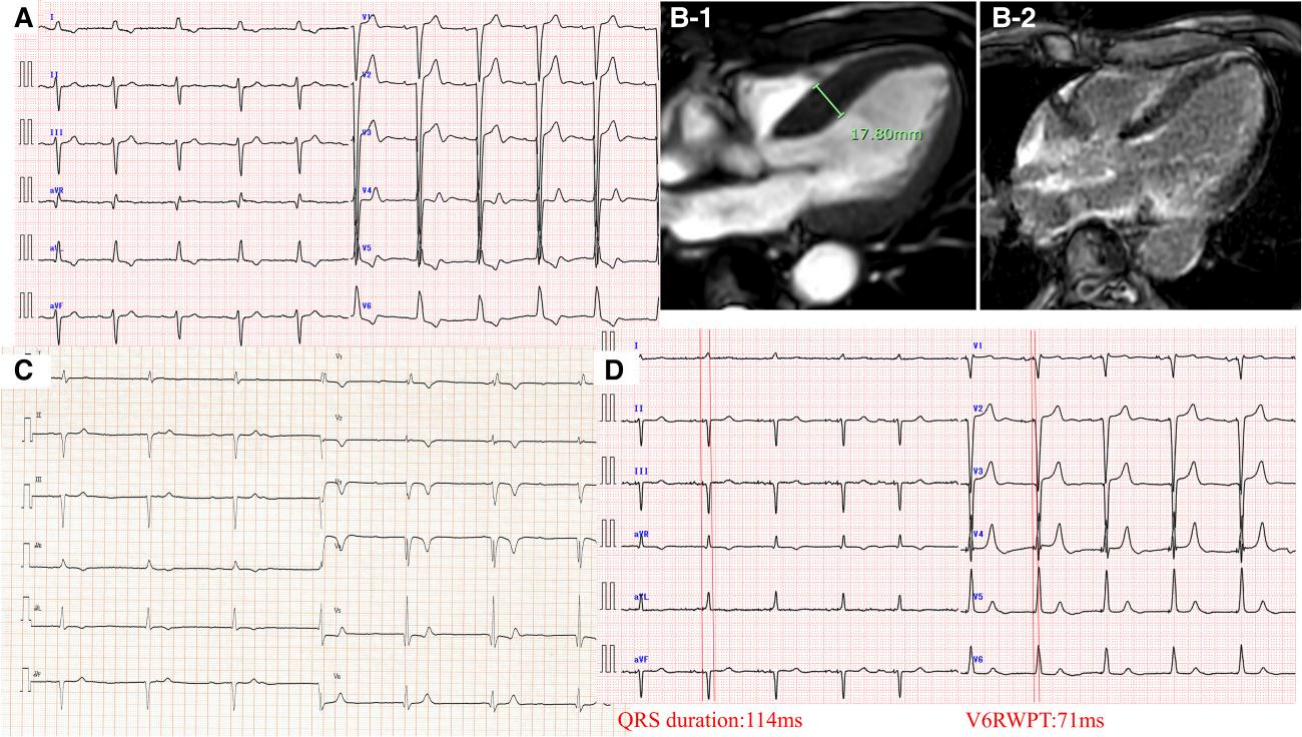
Patient 1—left bundle branch area pacing in wild-type transthyretin cardiac amyloidosis: electrocardiography and imaging insights before and after pacing. (*A*) Pre-pacemaker electrocardiography indicating a first-degree atrioventricular block with a complete left bundle branch block and a QRS duration of 142 ms. (*B-1*) Magnetic resonance imaging cine image showing left ventricular hypertrophy. (*B-2*) Late gadolinium enhancement magnetic resonance imaging image revealing significant endocardial enhancement indicative of diffuse fibrosis. (*C*) Subsequent electrocardiography showed progression to a third-degree atrioventricular block with a right bundle branch block-escape rhythm. (*D*) Post-pacemaker electrocardiography after left bundle branch area pacing demonstrating a reduced QRS duration to 114 ms with a V6 R-wave peak time of 71 ms and a right bundle branch block morphology at unipolar 1 V/0.4 ms. V6RWPT, V6 R-wave peak time.

**Figure 2:**
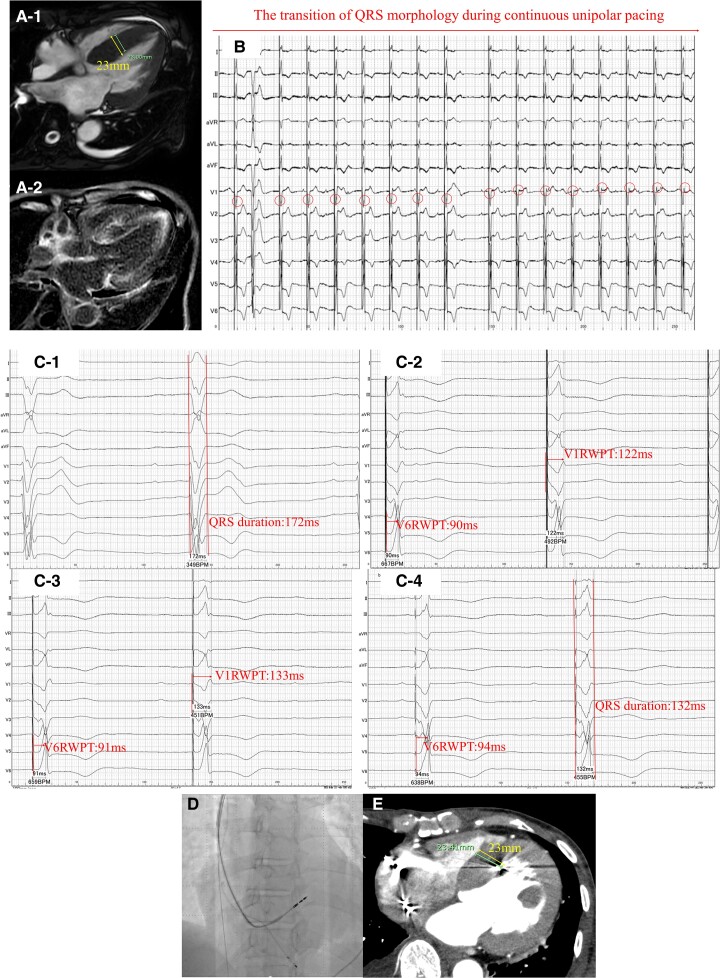
Patient 2—left bundle branch area pacing in hereditary transthyretin cardiac amyloidosis: an integrative pacing and imaging approach. (*A-1*) Magnetic resonance imaging cine image showing significant atrial septal hypertrophy with a septal thickness of 23 mm, indicating severe cardiac structural remodelling. (*A-2*) Magnetic resonance imaging demonstrating diffuse late gadolinium enhancement throughout the myocardium, consistent with extensive fibrosis typical of amyloid infiltration. (*B*) Continuous unipolar pacing electrocardiography tracing showing the transition of QRS morphology during left bundle branch area pacing. Note the progressive posterior shift of the notch and eventual development of an rSr’ pattern indicative of right bundle branch block. (*C-1*) Pre-implantation 12-lead electrocardiography demonstrating a wide QRS complex with a duration of 172 ms, characteristic of a complete left bundle branch block. (*C-2*) Intraoperative electrocardiography during threshold testing at unipolar 10 V/0.4 ms showing a V6 R-wave peak time of 90 ms and a V1 R-wave peak time of 122 ms, suggestive of non-selective left bundle branch pacing. (*C-3*) Intraoperative electrocardiography during threshold testing at unipolar 1 V/0.4 ms indicating a V6 R-wave peak time of 91 ms and a V1 R-wave peak time of 133 ms, with a V6-V1 interpeak of 42 ms, denoting selective left bundle branch pacing. (*C-4*) Post-implantation electrocardiography with bipolar 3 V/0.4 ms pacing demonstrates anodal capture with a narrowed QRS duration of 132 ms, confirming effective lead placement and pacing. (*D*) Fluoroscopic left anterior oblique view during the procedure showing the pacing lead deeply implanted within the septum due to the considerable septal thickness, a challenge usually encountered in patients with cardiac amyloidosis. An additional lead in the right ventricle is visible, which is a temporary pacing lead used during the procedure. (*E*) Post-procedure contrast-enhanced computed tomography verifies the precise placement of the pacing lead tip just beneath the left ventricular endocardium at the targeted site, ensuring optimal pacing lead function. V1RWPT, V1 R-wave peak time; V6RWPT, V6 R-wave peak time.

A 75-year-old male with a history of spinal canal stenosis presented with frequent syncope. On physical examination, the patient was alert with stable vital signs. Cardiovascular and pulmonary examinations were unremarkable, with no murmurs, gallops, crackles, or lower extremity oedema. Electrocardiography (ECG) revealed a first-degree AV block and complete left bundle branch block (LBBB) with a QRS duration of 142 ms, indicating significant electrical disturbance (*Figure 1A*). B-type natriuretic peptide (BNP) levels were elevated at 152.7 pg/mL. Transthoracic echocardiography showed concentric LV hypertrophy. Cardiac magnetic resonance imaging also demonstrated concentric LV hypertrophy with an interventricular septum thickness of 17 mm, confirming structural heart changes (*Figure 1B-1*) along with endocardial late gadolinium enhancement (LGE), suggesting the presence of diffuse fibrosis (*Figure 1B-2*) and delayed LV T1 values on native T1 mapping. Technetium pyrophosphate scan revealed diffuse Grade III uptake in the LV, indicating widespread amyloid deposition. A myocardial biopsy stained with Congo red and direct fast scarlet (DFS) confirmed amyloid deposition. The immunostaining result was positive for the transthyretin antibody, and ATTR genetic testing was negative, consistent with a diagnosis of wild-type ATTR-CA. The subsequent ECG, recorded 2 weeks after the initial hospitalization, showed progression to a third-degree AV block with a right bundle branch block (RBBB)-escape rhythm (*Figure 1C*), fulfilling the criteria for permanent pacemaker implantation.

Left bundle branch area pacing was performed using a SelectSecure 3830 pacing lead (Medtronic, Minneapolis, MN, USA) delivered via a fixed-curve C315-HIS sheath. The LBBP procedure was performed in accordance with previously established methods and criteria.^8^ Additionally, the ventricular lead was anchored to the septum and advanced towards the left side. Pacing led to a gradual narrowing of the QRS complex in lead V1, eventually forming a vertical R wave indicative of RBBB morphology (*Figure 1C*), and the V6 R-wave peak time (V6RWPT) remained consistently short across different outputs (unipolar 10 V/0.4 ms: 71 ms; unipolar 1 V/0.4 ms: 71 ms), achieving selective LBBP and reducing QRS duration to 114 ms (*Figure 1D*). These changes in ECG parameters reflected an improvement in electrical synchrony. Following pacemaker implantation, the patient was treated with tafamidis. Three years after the implantation and initiation of tafamidis, the patient did not experience heart failure. B-type natriuretic peptide levels decreased from 152.7 to 65.8 pg/mL, reflecting symptomatic and biomarker evidence of clinical stability. Before LBBAP, the patient’s LVEF was 64%, which remained well preserved at 60% during the follow-up period after LBBAP.

## Patient 2

A 64-year-old male with no significant past medical history and no known family history of heart disease presented with frequent syncope. He reported no prior episodes of palpitations, chest pain, or exertional dyspnoea. On physical examination, the patient was alert with stable vital signs. Cardiovascular and pulmonary examinations were normal, with no audible murmurs, crackles, or peripheral oedema. On admission, ECG showed a heart rate of 32 b.p.m. with common atrial flutter (AFL). The baseline ECG revealed a complete LBBB with a wide QRS duration of 172 ms (*Figure 2C-1*). Transthoracic echocardiography revealed preserved ventricular contraction but significant hypertrophy with a septal thickness of 20 mm (*Figure 2A-1*). Cardiac magnetic resonance imaging revealed diffuse LGE (*Figure 2A-2*) with a maximum wall thickness of 23 mm. Technetium pyrophosphate scintigraphy indicated diffuse uptake in the LV wall with a Perugini classification of +3 and a heart-to-contralateral lung (H/CL) ratio of 2.20. Myocardial biopsy with Congo red and DFS staining showed positive results. Genetic testing revealed a Val30Met mutation, confirming hereditary ATTR-CA.

The treatment plan included ablation, pacemaker implantation, and patisiran administration. Owing to the presence of an AFL dependent on the tricuspid valve-inferior vena cava isthmus, ablation was performed on this area. After ablation, a complete AV block was observed on the ECG, necessitating the implementation of LBBAP. Left bundle branch area pacing was performed using a Solia S pacing lead and Selectra 3D (Biotronik, Berlin, Germany) with continuous pacing from the stylet, owing to significant cardiac hypertrophy.

**Figure 3:**
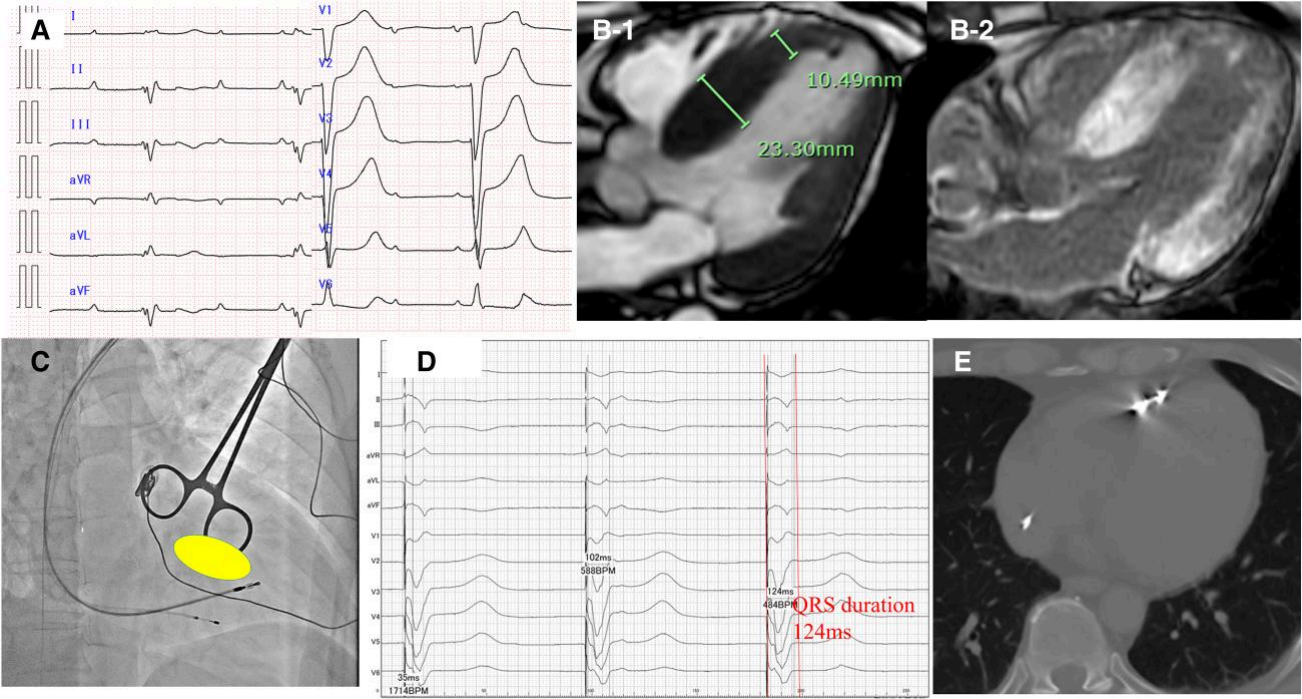
Patient 3—integrative diagnostic and therapeutic imaging in hereditary transthyretin cardiac amyloidosis. (*A*) Pre-pacemaker electrocardiography showing a complete atrioventricular block with a left bundle branch block escape rhythm, characterized by a QRS duration of 165 ms. (*B-1*) Cardiac magnetic resonance imaging cine image shows non-uniform hypertrophy of the ventricular septum, with substantial thickening at the base and mid-portion (23 mm) and no hypertrophy at the apical side (10 mm). (*B-2*) Cardiac magnetic resonance imaging late gadolinium enhancement image showing strong enhancement in the hypertrophied regions of the septum, indicative of amyloid deposition, with less enhancement in the apical region. (*C*) Fluoroscopic right anterior oblique view during left bundle branch area pacing highlighting areas with high pacing thresholds (enclosed by ellipses) unsuitable for lead placement, necessitating alternative site selection. (*D*) Post-pacemaker electrocardiography during left bundle branch area pacing at bipolar 3.5 V/0.4 ms showing anodal capture with a QRS duration of 124 ms and a deep S wave in the V6 lead, suggesting successful left bundle branch area pacing. However, the relatively short peak left ventricular activation time of 42 ms observed in V6 is likely due to the lead’s placement closer to the apical region, and it may not fully reflect the complete left ventricular activation time. Despite this, the right bundle branch block pattern in V1 strongly supports successful left bundle branch area pacing. (*E*) Postoperative plain computed tomography confirms precise lead placement at the targeted site, corresponding to areas of thinner myocardium and less late gadolinium enhancement identified on magnetic resonance imaging.

During the procedure, the transition of the QRS morphology during continuous unipolar pacing was monitored, and the pacing lead was adjusted until a typical RBBB pattern appeared in V1, indicating successful left bundle capture (*Figure 2B*). Pre-implantation ECG showed a complete LBBB with a QRS duration of 172 ms (*Figure 2C-1*). With unipolar 10 V/0.4 ms pacing, the V6RWPT was 90 ms, and the V1 R-wave peak time (V1RWPT) was 122 ms, suggesting non-selective LBBP (*Figure 2C-2*). In contrast, with unipolar 1 V/0.4 ms pacing, the V6RWPT remained unchanged at 91 ms, and the V1RWPT was prolonged to 133 ms, with a V6-V1 interpeak of 42 ms, indicating selective LBBP (*Figure 2C-3*). Anodal capture resulted in a QRS duration of 132 ms with bipolar 3 V/0.4 ms (*Figure 2C-4*).

Fluoroscopic imaging of the left anterior oblique view during the procedure revealed a pacing lead deeply implanted within the thickened septum, which is usually challenging in patients with cardiac amyloidosis (*Figure 2D*). An additional lead visible in the RV was a temporary pacing lead used during the procedure. Post-procedural contrast-enhanced computed tomography (CT) confirmed the precise location of the lead tip immediately beneath the endocardium of the targeted LV area (*Figure 2E*). Following pacemaker implantation, patisiran treatment was initiated. Two years after the implantation and commencement of patisiran, the patient did not experience heart failure. Over a 2-year follow-up, BNP levels decreased from 210.1 to 93.2 pg/mL.

## Patient 3

A 59-year-old male with a history of hypertension and no known family history of heart disease presented with progressive dyspnoea on exertion. He denied experiencing prior episodes of syncope, chest pain, or palpitations. On physical examination, the patient was alert with stable vital signs. Cardiovascular and pulmonary examinations revealed no audible murmurs or crackles. However, bilateral lower extremity oedema and numbness in both hands were noted. Electrocardiography showed left-axis deviation with a complete RBBB. Cardiac magnetic resonance imaging revealed heterogeneous LV hypertrophy, with a septal thickness measuring 23 mm from the base to the mid-portion and 10 mm at the apical side (*Figure 3B-1*). Late gadolinium enhancement matched the hypertrophy, which was more pronounced in the thicker parts of the septum and less pronounced on the apical side (*Figure 3B-2*). The extracellular volume fraction increased to 62.5%, indicating substantial myocardial involvement.

Technetium pyrophosphate scintigraphy revealed diffuse uptake in the LV wall with a Perugini score of +3 and a H/CL ratio of 2.44, consistent with significant amyloid deposition. A myocardial biopsy with Congo red and DFS staining confirmed the presence of amyloids. Genetic testing revealed a Pro24Ser mutation, confirming a diagnosis of hereditary ATTR-CA.

Approximately 2 years after starting treatment with patisiran, the patient developed a complete AV block. The pre-pacemaker ECG showed a QRS duration of 165 ms, consistent with an LBBB escape rhythm (*Figure 3A*). Left bundle branch area pacing was performed using the SSPC + Ingevity system (Boston Scientific, Marlborough, MA, USA) to manage this condition. During the procedure, the right anterior oblique cine image revealed regions with significantly high threshold levels (marked in yellow) unsuitable for lead placement. Consequently, the lead was placed closer to the apical side, where the myocardium was thinner and LGE was less intense (*Figure 3C*). Pacing at bipolar 3.5 V/0.4 ms showed anodal capture with a QRS duration of 124 ms and a deep S wave in the V6 lead, suggesting successful apical LBBAP (*Figure 3D*). However, the relatively short peak LV activation time of 42 ms in V6 is likely due to the lead’s placement closer to the apex, which may result in faster myocardial activation that does not fully reflect the left bundle branch capture. Despite this, the RBBB pattern observed in V1 strongly supports successful LBBAP, indicating effective pacing within the left bundle branch area. A post-procedural plain CT scan confirmed precise lead placement in the targeted thinner myocardium and less LGE area (*Figure 3E*). Since the LBBAP, the patient has not experienced heart failure progression, and his exertional dyspnoea has improved. Before LBBAP, the patient’s LVEF was 52%, and it remained stable at 52% during the follow-up period after LBBAP.

## Summary

The baseline characteristics, procedural details, and follow-up data for each patient are summarized in Summary figure. This table provides a comprehensive overview of the clinical and procedural details for each patient, providing context for the results and discussion that follow.

## Discussion

The cases presented here underscore the multifaceted challenges in managing ATTR-CA, particularly when complicated by conduction system disease necessitating pacing interventions. While adopting LBBAP has emerged as a promising strategy, providing advantages over traditional pacing methods, particularly in the context of cardiac amyloidosis, where myocardial infiltration can significantly alter tissue conduction, it is not without its complexities.

A shared difficulty across all three patients was the presence of significant interventricular septal hypertrophy, a hallmark of ATTR-CA. This hypertrophy requires the pacing lead to be inserted deep into the septum to reach the left bundle area, a procedure that carries the risk of perforating the LV cavity. Recent studies highlight the importance of meticulous fluoroscopic guidance and advanced technical skills in managing these anatomical challenges.^4^ Magnetic resonance imaging plays a crucial role in this context by allowing precise identification of scar tissue locations, which can help guide lead placement and minimize the risk of complications such as perforation.

Additionally, the presence of myocardial scarring can further complicate the implantation process. Scarring may lead to unstable electrical contact, resulting in increased pacing thresholds and unstable lead impedance. Recent studies have discussed these challenges, noting that in the presence of scarring, the lead’s stability and electrical performance can be compromised, necessitating careful selection of the pacing site and lead type.^9^ Moreover, the use of MRI to accurately map these scar locations before the procedure can enhance the safety and efficacy of LBBAP by ensuring the lead is placed in viable myocardial tissue.

To further reduce the risk of LV perforation, the application of continuous pacing techniques during lead implantation has proven useful.^10^ This method allows for real-time monitoring of electrical parameters, helping to ensure that the lead remains within the desired myocardial area without inadvertently advancing into the LV cavity. Continuous pacing techniques not only provide continuous feedback during the lead placement but also help in managing septal hypertrophy and scarring more effectively.

The selection of the appropriate lead and catheter is also critical in overcoming these challenges. Recent studies compare the use of stylet-driven leads (SDLs) and lumenless leads (LLLs) for LBBAP.^11^ Stylet-driven leads provide greater stiffness, facilitating advancement through a thickened septum, while LLLs allow more precise positioning, which is essential for optimal pacing outcomes. The choice of lead should be tailored to the patient’s anatomy and the degree of myocardial hypertrophy.

In our cases, we customized the selection of pacing leads based on each patient’s unique anatomical and pathological characteristics, guided by pre-procedural imaging and intraoperative evaluation. For Patient 1, who exhibited moderate interventricular septal hypertrophy with relatively preserved myocardial tissue, we selected the SelectSecure 3830 lead due to its flexibility and ability to reach deep within the septum while ensuring precise LBB capture, using the C315-HIS delivery sheath for enhanced manoeuvrability. In Patient 2, whose severe septal hypertrophy (septal thickness of 23 mm) made deep septal penetration challenging, we used the Solia S lead with the Selectra 3D delivery sheath, offering superior pushability and stable fixation. Additionally, continuous advancement using a SDL reduced the risk of myocardial perforation while maintaining electrical contact. In Patient 3, who had asymmetric septal hypertrophy and extensive myocardial infiltration caused by ATTR-CA, we opted for a SSPC sheath, which is flexible and manoeuvrable within the ventricular cavity, enabling precise lead placement in minimally scarred regions. Furthermore, the configuration supported stylet-guided continuous pacing, allowing real-time assessment of electrical parameters and ensuring safe and stable lead fixation during the procedure.

Cases such as Patient 2, characterized by pronounced myocardial hypertrophy and interventricular conduction delay leading to prolonged V6RWPT, and Patient 3, marked by heterogeneous hypertrophy and amyloid infiltration resulting in high pacing thresholds, exemplify these technical challenges. These scenarios highlight the necessity for nuanced pacing strategies in the presence of amyloidosis-related AV blocks.

Importantly, the V6-V1 interpeak interval has emerged as a valuable diagnostic criterion when V6RWPT alone may be unreliable.^12^ Conditions such as intraventricular conduction delay or LBBB can cause prolonged V6RWPT even when left bundle branch capture is present. In such cases, the V6-V1 interpeak interval remains relatively unaffected because it reflects proportional delays in RV and LV activation. Applying this criterion alongside V6RWPT improves diagnostic accuracy and supports precise assessment of LBBAP.

The potential for overcoming these technical hurdles exists. Electrophysiological insights, such as the evaluation of V6-V1 interpeak intervals, along with the application of techniques, such as continuous pacing, the use of advanced imaging, including MRI, and careful selection of various delivery catheters,^9,11,13^ provide viable solutions. Mastery of these specialized techniques is paramount for navigating the complexities of LBBAP in patients with significant myocardial alterations caused by amyloid infiltration.

Moreover, recent studies further corroborate the feasibility and safety of LBBAP in patients with cardiac amyloidosis, emphasizing its potential as a viable pacing strategy for this complex patient population.^4^ The evidence from Patient 1, demonstrating improved electrical synchrony through LBBAP, as evidenced by reduced QRS duration and BNP levels, along with the absence of heart failure development over a 3-year follow-up, underscores the long-term benefits of LBBAP in managing cardiac amyloidosis with concomitant conduction disease.

## Lead author biography


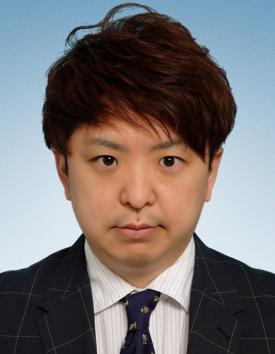


Dr Keisuke Miyajima graduated Hamamatsu University School of Medicine in 2008 and began his medical training at the Hamamatsu University Medical Center. He then continued his practical training in the field of cardiology and is currently working as a cardiologist at the Seirei Mikatahara General Hospital.

## Data Availability

Raw data were generated at Seirei Mikatahara General Hospital. Derived data supporting the findings of this study are available from the corresponding author, K.M., upon request.

## Notes

**Consent:** The authors confirm that written consent for the submission and publication of this case series, including images and associated text, has been obtained from the patients in line with COPE guidance.

**Funding:** This case series did not receive any specific grant from funding agencies in the public, commercial, or not-for-profit sectors.
